# Influence of Vitamin C Supplementation on Oxidative Stress and Neutrophil Inflammatory Response in Acute and Regular Exercise

**DOI:** 10.1155/2015/295497

**Published:** 2015-02-23

**Authors:** Ljiljana M. Popovic, Nebojsa R. Mitic, Dijana Miric, Boban Bisevac, Mirjana Miric, Brankica Popovic

**Affiliations:** ^1^Institute of Pathophysiology, Medical Faculty Pristina, 38220 Kosovska Mitrovica, Serbia; ^2^Institute of Biochemistry, Medical Faculty Pristina, 38220 Kosovska Mitrovica, Serbia; ^3^Institute of Physiology, Medical Faculty Pristina, 38220 Kosovska Mitrovica, Serbia; ^4^Department of Informatics and Computer Sciences, Academy of Criminalistic and Police Studies, Cara Dusana 196, 11080 Belgrade, Serbia

## Abstract

Exercise induces a multitude of physiological and biochemical changes in blood affecting its redox status. Tissue damage resulting from exercise induces activation of inflammatory cells followed by the increased activity of myeloperoxidase (MPO) in circulation. Vitamin C readily scavenges free radicals and may thereby prevent oxidative damage of important biological macromolecules. The aim of this study was to examine the effect of vitamin C supplementation on oxidative stress and neutrophil inflammatory response induced by acute and regular exercise. Experiment was conducted on acute exercise group (performing Bruce Treadmill Protocol (BTP)) and regular training group. Markers of lipid peroxidation, malondialdehyde (MDA), MPO activity, and vitamin C status were estimated at rest and after BTP (acute exercise group) and before and after vitamin C supplementation in both groups. Our results showed increased postexercise Asc in serum independently of vitamin supplementation. They also showed that vitamin C can significantly decrease postexercise MDA level in both experimental groups. Increased postexercise MPO activity has been found in both groups and was not affected by vitamin C supplementation. We concluded that vitamin C supplementation can suppress lipid peroxidation process during exercise but cannot affect neutrophil inflammatory response in either exercise group.

## 1. Introduction

Increased interest in regular physical activity, oxidative stress, and the importance of antioxidants is probably due to the modern concept of healthy lifestyle. However, evidences suggest that though exercise can help health improvement, vigorous physical activity may increase blood temperature and lactate and decrease blood pH and partial oxygen pressure, causing disruption of homeostasis and impairment of blood redox status [1]. The contracting skeletal muscle can generate high levels of free radicals; thus the prolonged and intense exercise can result in oxidative damage of macromolecules in both blood and skeletal muscle, leading to oxidative stress. On the other side, low to moderate levels of oxidants probably play significant regulatory roles, such as control of gene expression, regulation of cell signaling pathways, and modulation of skeletal muscle force production [2, 3].

Exhaustive exercise, especially when sporadic, can produce muscle damage and initiate an acute inflammatory response, evidenced by increased cytosolic enzymes in the blood, and sarcolemma and *Z* line disruption [4]. Muscle soreness, which occurs after such activities, is considered as a consequence of microvascular disruption, edema, and cell damage induced by mechanical force or disturbance of normal cellular metabolism [5]. Inflammation and muscle injury are usually accompanied by rapid invasion of macrophages and other inflammatory cells, lasting days to weeks while muscle repair and regeneration occur [6, 7].

During exercise activated phagocytes can serve as important source of free radicals and other oxidants. Superoxide anion radical, formed by activated leukocyte NADPH oxidase, is enzymatically or spontaneously converted into hydrogen peroxide (H_2_O_2_), which is an oxidizing substrate for myeloperoxidase (MPO), enzyme secreted by activated phagocytes [6]. MPO released from leukocytes can catalyze the formation of a number of oxidants contributing to sustained tissue damage, including the degradation of extracellular matrix collagen and proteoglycans [8].

Growing body of evidence suggests that antioxidant supplementation can be a useful strategy to reduce harmful effects of exercise induced oxidative stress [9]. Vitamin C is the major water-soluble antioxidant present within the cell and extracellular fluids. This vitamin is able to provide protection against phagocyte-derived oxidants by reducing the adhesion of phagocytes to endothelium, attenuating respiratory burst, and preventing subsequent lipid peroxidation [10]. Given that diet is the main source of vitamin C in humans, the aim of this study was to examine the effect of vitamin C supplementation on oxidative stress and neutrophil inflammatory response in acute and regular exercise.

## 2. Materials and Methods

### 2.1. Subjects

This study included two experimental groups: acute exercise group and regular training group. Acute exercise group consisted of 30 healthy sedentary male volunteers, with average age 22.5 ± 1.5 years, body weight 70.6 ± 7.2 kg, and height 175.6 ± 6.3 cm. Regular training group included 30 professional male athletes, with average age 24.5 ± 2.5 years, weight 76.6 ± 3.2 kg, and height 180.2 ± 3.1 cm, who were on regular training for minimum 4 years. All subjects were nonsmokers and not on vitamin, mineral, or other medications known to affect oxidative stress markers. In order to minimize the effect of diurnal biological variations, each subject was tested at the same time of the day. Participants were informed about the experimental protocol and possible risks, and all subjects gave their consent. The study was conducted in accordance with the Declaration of Helsinki and ethical clearance for the study was obtained from the Ethical Committee of Medical Faculty Pristina, Kosovska Mitrovica.

### 2.2. Experimental Protocol

As a model for acute exercise we used Bruce Treadmill Protocol (BTP) [11]. Participants run on the treadmill for exhaustive running exercise test. Heart rate was monitored continuously during BTP. The criterion for ending exhaustive exercise was achievement of 80% of age predicted maximal heart rate, calculated as 210 − age (in years). No consumption of liquids was allowed during BTP. Participants in regular training group did not make any changes in their daily routine.

Both exercise groups were supplemented with 2 grams of vitamin C per day. This dose was selected as a daily tolerable upper intake level in order to prevent diarrhea and gastrointestinal disturbances [12]. Vitamin C was taken orally for 14 days, 4 times a day in a 500 mg dose.

In acute exercise group blood samples were collected before and after vitamin C supplementation both at rest and after BTP (total of 4 measurements). In regular training group, who kept their prerecruitment protocol and normally trained twice a day, the blood was taken before and after supplementation period (total of 2 measurements).

### 2.3. Biochemical Methods

Venous blood (5 mL) was taken into a vacutainer tube without anticoagulant and spun after 30 minutes. Only clear, hemolysis-free samples were included in the study and analyzed daily. All biochemical measurements were carried out on an UV/VIS spectrophotometer equipped with a constant temperature cuvette compartment (SAFAS 2, Monaco).

#### 2.3.1. Assessment of Malondialdehyde

Concentration of malondialdehyde (MDA), a product of lipid peroxidation and oxidative stress biomarker, was assessed spectrophotometrically as a thiobarbituric acid reactive substance [13]. The absorbance readings were taken at 512, 532, and 552 nm against reagent blank, and Allen's correction was applied. Concentration of MDA was calculated using the corrected absorbance at *λ* = 532 nm and the molar absorbance of Trimetin complex of 1.56 × 10^5^ × L × M^−1^ × cm^−1^.

#### 2.3.2. Determination of Reduced and Oxidized Vitamin C

Vitamin C is present in the serum as a reduced form, or ascorbic acid (Asc), and as oxidized vitamin form, consisted mostly of dehydroascorbic acid (DHA). Total vitamin C was determined by 2,4-dinitrophenylhydrazine (DNPH) method [14], after sample deproteinization (m-phosphoric acid, 60 g/L; EDTA, 2 mM). In this method, Asc present in the sample is firstly oxidized by copper(II) sulfate to DHA. Thereafter, preexisting and newly formed DHA reacts under acidic conditions with DNPH reagent (thiourea, 32.85 mmol/L; copper (II) sulfate, 1.88 mmol/L; 2,4-dinitrophenylhydrazine, 90 mmol/L; and sulfuric acid, 4.05 mol/L) to give red colored hydrazone complex.

For determination of preexisting DHA we used a DNPH reagent in which copper(II) sulfate was omitted. The absorbance readings were taken at *λ* = 520 nm against reagent blank. Freshly prepared solutions of ascorbic acid in the concentration range of 0–120 *μ*mol/L were used to construct the calibration curve. The concentration of Asc was calculated as the difference between total vitamin C and preexisting DHA.

#### 2.3.3. Measurement of Myeloperoxidase Activity

Serum MPO activity was determined by modified Trinder's reaction [15], using sodium azide to block catalase activity. Briefly, 1.3 mL reaction mixture (3.08 mmol/L 4-aminoantipyrine, 0.215 mol/L phenol, and 1.2 mmol/L sodium azide) was mixed with 1.5 mL freshly prepared hydrogen peroxide (1.7 mmol/L) and incubated for 5 min at 25°C. After addition of 0.1 mL serum the formation of quinoneimine was monitored at *λ* = 505 nm (25°C) for 3 min. One unit of MPO activity was defined as an amount of enzyme degrading 1 *μ*mol of hydrogen peroxide per minute and calculated using a molar absorbance of quinoneimine of 1.3 × 10^4^ × L × M^−1^ × cm^−1^.

### 2.4. Statistical Methods

The statistical analysis was performed using a commercial software package (SPSS version 12.0 for Windows, SPSS Inc., IL, USA). The data are reported as mean value ± SD. The statistical procedures included paired-samples Student's *t*-test, Wilcoxon signed-rank test, and Mann-Whitney test. Differences were considered significant at *P* < 0.05.

## 3. Results

Comparisons of presupplemental serum oxidative stress markers and vitamin C status between acute exercise and regular training groups are presented in Table 1. In comparison to acute exercise group, serum MDA, vitamin C, DHA/Asc, and MPO activity were significantly higher in regular training group, pointing to persistent lipid peroxidation, neutrophil inflammatory response, and participation of vitamin C in antioxidant defense mechanisms.

**Figure 1:**
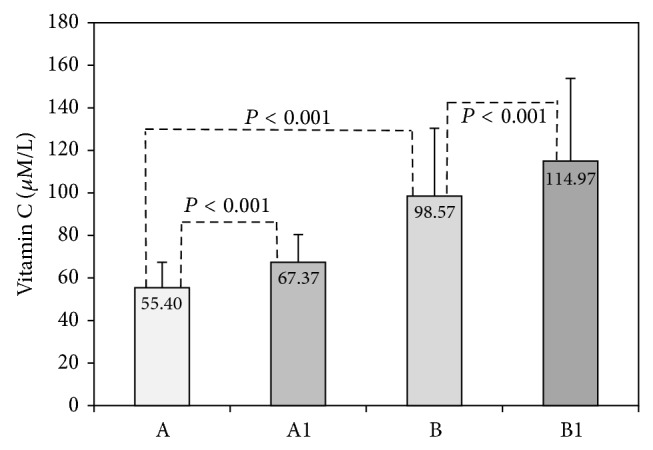
Serum vitamin C in acute exercise group. Concentrations of total vitamin C were measured before vitamin C supplementation in rest (A) and after BTP (A1) and after 14 days of vitamin C supplementation in rest (B) and after BTP (B1).

**Figure 2:**
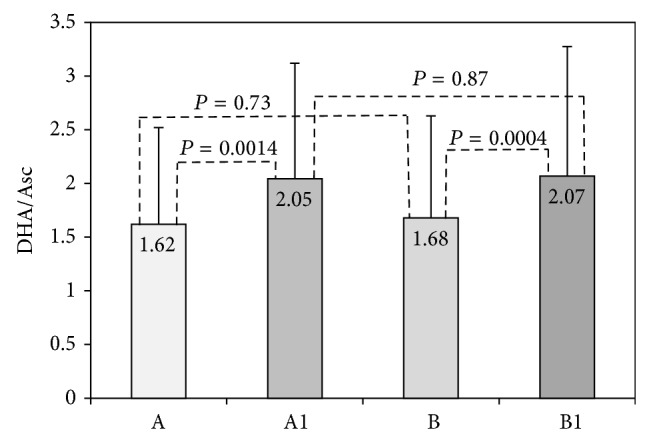
DHA/Asc ratio in acute exercise group. DHA/Asc ratio was determined before vitamin C supplementation in rest (A) and after BTP (A1) and after 14 days of vitamin C supplementation in rest (B) and after BTP (B1).

**Figure 3:**
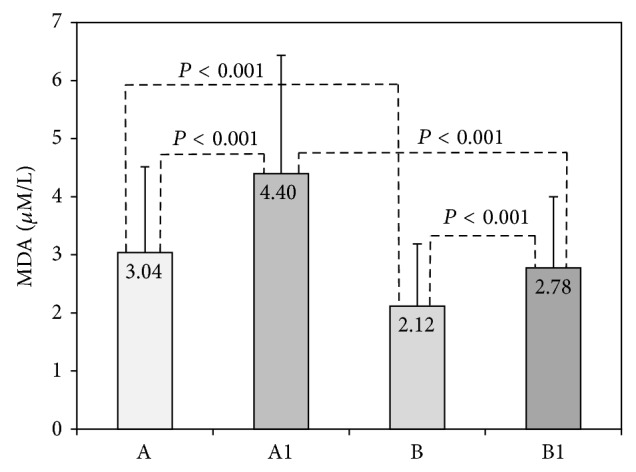
Malondialdehyde in acute exercise group. Malondialdehyde (MDA) was measured before vitamin C supplementation in rest (A) and after BTP (A1) and after 14 days of vitamin C supplementation in rest (B) and after BTP (B1).

**Figure 4:**
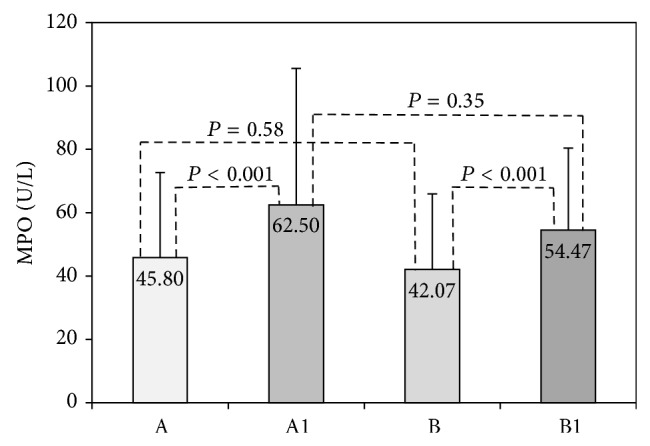
Myeloperoxidase activity in acute exercise group. Myeloperoxidase (MPO) activity was measured in serum before vitamin C supplementation in rest (A) and after BTP (A1), and after 14 days vitamin C supplementation in rest (B) and after BTP (B1).

Results obtained in acute exercise group are shown in Figures 1–4. Changes of serum vitamin C in acute exercise group are presented in Figure 1. As can be seen, vitamin C basal levels increased from initial 55.40 *μ*mol/L to 98.57 *μ*mol/L after 14 days of supplementation, indicating that participants complied with given experimental protocol. We also observed that postexercise vitamin C increased independently of supplementation (55.40 versus 67.37 *μ*mol/L before and 98.57 versus 114.97 *μ*mol/L after supplementation). Both changes were statistically significant (*P* < 0.001).

We further estimated antioxidant consumption of vitamin C using the ratio of DHA and Asc (Figure 2). After BTP the DHA/Asc ratio increased, and the difference from basal levels was significant both in samples taken before (1.62 versus 2.05; *P* < 0.05) and after vitamin C supplementation (1.68 versus 2.97; *P* < 0.05). Still, there was no significant difference between postexercise values before and after vitamin C supplementation (2.05 versus 2.07; *P* = 0.87), probably because the same exercise test (BTP) was applied.

Changes of serum MDA, as a marker of lipid peroxidation, are shown in Figure 3. Acute exercise significantly increased serum MDA before (3.04 versus 4.40 *μ*mol/L, *P* < 0.001) and after vitamin C supplementation (2.12 versus 2.78 *μ*mol/L, *P* < 0.001). In comparison to initial levels serum MDA significantly decreased after vitamin C supplementation in preexercise (3.04 versus 2.12 *μ*mol/L; *P* < 0.05) as well as in postexercise samples (4.40 versus 2.78 *μ*mol/L; *P* < 0.001).

Serum MPO activity was assessed as a marker of neutrophil inflammatory response and degranulation (Figure 4). Our results showed a significant increase in postexercise serum MPO activity, independently of vitamin C supplementation. The BTP induced a significant increase in MPO activity from basal 45.8 U/L to 62.5 U/L in nonsupplemented and from 42.07 U/L to 54.47 U/L in samples obtained after vitamin C supplementation. Preexercise serum MPO activities (45.80 versus 42.07 U/L; *P* = 0.58), as well as postexercise MPO activities (62.50 versus 54.47 U/L, *P* = 0.35), were similar regardless of vitamin C supplementation (Figure 4).

Results presenting changes of MDA, total vitamin C and its redox status, and MPO activity induced by vitamin C supplementation in regular training group are summarized in Table 2.

Similarly to acute exercise group, serum MDA decreased in regular training group after 14 days of vitamin C supplementation from 4.26 to 3.3 *μ*mol/L (*P* < 0.001). On the other side, the DHA/Asc ratio was not changed by supplementation (2.16 versus 1.99; *P* = 0.14), pointing that training intensity remained unchanged during experimental protocol. There was also no significant difference in serum MPO activity before and after supplementation (68.83 versus 71.07 U/L; *P* = 0.45), suggesting that vitamin C supplementation had no effect on neutrophil inflammatory response in regular training group.

## 4. Discussion

During intense exercise skeletal muscles endure mechanical and metabolic changes, which in most other body cells induce serious injury. Indeed, there is no evidence that any other tissue experiencing such drastic changes in oxygen metabolism during activities considered as “normal” [16]. It has been estimated that resting energy expenditure for skeletal muscle is about 13 kcal/kg org mass/day, but during heavy exercise it can increase more than 100 times. This process is associated with increased need for ATP and enhanced aerobic and/or anaerobic metabolism, resulting in increased free radical production and subsequent oxidative stress [3]. Membrane-bound polyunsaturated fatty acids attacked by free radicals undergo lipid peroxidation, accompanied by generation of new, highly destructive free radicals, spreading the reaction. Intensity of lipid peroxidation is usually quantified by measuring MDA [17].

Numerous studies have demonstrated changes in lipid peroxidation following exercise. Depending on exercise protocol, serum MDA has been reported to be increased [1, 18–21], but other studies reported different responses depending on exercise intensity [22, 23]. Also, there was no or little change in blood MDA level following exercise [24, 25]. Our results (Figure 3, Table 2) showed that both acute and regular exercise significantly increased serum MDA levels, thereby confirming the role of exercise in free radical production and consequent oxidative stress. Lipid peroxidation can cause decrease in cell membrane fluidity, inability to maintain ionic gradient, cellular swelling, and tissue inflammation. All these changes modulate a variety of cellular processes, eventually leading to limited muscle contraction [26]. Considering the inconsistency of the literature data we believe that there is still a need for further research of lipid peroxidation process and exercise-induced oxidative damage.

It has been previously noted that high intensity exercise (>60% max oxygen consumption) and eccentric and long duration exercise are often accompanied by tissue damage and local inflammation [27]. Increased muscle loading or injury produces an early increase of MPO activity in muscles and circulation, indicating neutrophil invasion and degranulation [6]. Neutrophil functions during and after physical activity have been extensively studied. It has been shown that mobilization of peripheral leukocytes occurs during acute exercise proportionally to intensity and duration of exercise. Mobilization of neutrophils is thought to be the result of selected redistribution from marginated pool, caused by increased stress-hormone levels after exercise [28]. Moreover, leukocyte extravasation can be directly correlated with tissue vascular supply [29]. Sureda et al. reported that exercise can also induce inflammation-like changes in immune cell count and acute phase protein release. They found that after exhaustive exercise circulating neutrophil count increased while lymphocytes rapidly decreased and that these changes lasted for several hours [30]. Further, Suzuki et al. reported that exhaustive exercise induced elevation of neutrophil cell count, associated with the duration or intensity of exercise, and accompanied by increased production of hypochlorous acid [31]. Similar results were obtained by other researchers investigating the role of neutrophils in exercise-induced oxidative stress [32].

In the current study, both acute and regular exercise significantly increased serum MPO activity (Figure 4, Table 2), which is in accordance to Morozov et al. [33]. These authors also reported that after swimming to exhaustion MPO neutrophil content decreased, but during recovery it returned to initial values in both plasma and neutrophils. They noticed significantly increased MPO content in human plasma immediately after exercise and after one hour of recovery period. Using a treadmill running until exhaustion test they found a close relationship between intensity of neutrophil degranulation and work capacity of the athletes [33].

Our results support findings of Sureda et al. [30] that there is a direct relationship between MPO activity and plasma MDA levels, pointing to the importance of neutrophils as a source of free radicals, contributing to lipid peroxidation process. In the current study serum MPO activity at rest was higher in regular training group compared with sedentary one (Table 1). This is in accordance with Morozov et al. who reported higher plasma MPO in trained rats [29]. In that study, MPO significantly increased after exercise both in untrained and trained animals [29]. Extracellular MPO can be powerful catalyst of lipid peroxidation process and readily induce chlorination and nitrosylation of various blood compounds, consequently producing a variety of dysfunctional molecules, toxic mediators, atherogenic lipids, and oxidized protein products [34]. It has been suggested that some antioxidants, such as vitamin E and estradiol, may enhance the MPO catalyzed oxidation, indicating that these antioxidants could actually act as prooxidants in the presence of MPO [35]. Some recent data also suggest that higher MPO concentration may predict the development of coronary events in apparently healthy people with evidence of subclinical atherosclerotic plaque [8].

Nonenzymatic low-molecular-weight antioxidants, like vitamins and uric acid, are important players in blood antioxidant defense mechanisms. These antioxidants act either forming less reactive radicals or quenching the free radical reaction. Vitamin C is a hydrophilic antioxidant and because pKa of ascorbic acid is 4.25 the ascorbate anion is the predominant form existing at physiological pH in the blood. Vitamin C can act directly scavenging superoxide, hydroxyl, and lipid hydroperoxide radicals or, indirectly, playing an important role in recycling of vitamin E [2]. The most striking chemical activity of ascorbic acid is its ability to act as a reducing agent, implicated in detoxifying various oxygen radicals in vivo. The donation of one electron by Asc produces semidehydroascorbate radical, which can be further oxidized to DHA. The change in DHA/Asc ratio is commonly used for estimation of antioxidant role of ascorbic acid. Although sport population (both professionals and recreationists) heavily utilize antioxidant supplementation, there is no clear evidence whether it is beneficial for muscle function. There are numerous studies reporting that antioxidant supplementation can delay muscle fatigue during submaximal contraction, but it seems that antioxidants are poorly effective if muscle contraction is near maximum, and can barely improve endurance exercise performance or faster recovery time [2]. A clear example of protective effect of an antioxidant has been shown when allopurinol was administrated before cyclist competition in the Tour de France, by attenuating the increase of serum creatine kinase and aspartate aminotransferase [4].

In our study increased postexercise serum Asc concentration was independent of vitamin C supplementation (Figure 1). This is in accordance to Bailey et al. who reported a strong positive response of plasma vitamin C and cortisol to exercise, indicating an endogenous release of vitamin C from adrenal glands, concomitantly with cortisol release [36]. Vollaard et al. [5] noticed that increased postexercise plasma ascorbic acid rapidly returned to basal value and dropped even below one day after exercise, remaining low for at least 3 days. The same authors concluded that the rise of plasma antioxidant levels may enhance antioxidant defense of the blood but would probably weaken the defense at the site where mobilized from. We also noticed that vitamin C supplementation decreased serum MDA in regular training group as well as in acute exercise group (Table 2, Figure 3). These results suggest that higher levels of vitamin C can attenuate but cannot completely prevent lipid peroxidation process induced by exercise. Sureda et al. [37] reported that supplementation (152 mg/day vitamin C and 50 mg/day vitamin E) significantly increased basal neutrophil vitamin C compared with placebo and that exercise decreased vitamin C concentration in both groups after recovery. They concluded that antioxidant supplementation with vitamin C reduced exercise-induced oxidation of proteins and neutrophils. Close et al. reported that ascorbic acid supplementation (1 g) caused increase in plasma ascorbate concentration, sufficient to deal with increased free radical production and prevent lipid peroxidation and formation of MDA [10].

Examining the effect of ascorbic acid supplementation on neutrophil inflammatory response we found no significant change in MPO activities in both types of exercise (Figure 4, Table 2). These results are in agreement with Gaut et al. [38]. In a mouse model of acute inflammation, these authors provided evidences that ascorbate could not either directly or indirectly block the oxidation of amino acids and lipids by free radicals derived from phagocyte NADPH oxidase. Similar results were reported by Bohlooli et al. [39] who showed that acute moderate doses of vitamin C affected exercise-induced lipid peroxidation and muscle damage but had no influence on inflammatory markers. They also reported significantly increased total leukocytes, neutrophils, CRP, and IL-6 in response to exercise.

We also compared basal values of serum MDA in acute exercise group (sedentary voluntaries) and regular training group (professional athletes) as shown in Table 1. We noted that even in the state of rest a regular training group had higher serum MDA than acute exercise group. These results point that regular training process is inevitably accompanied by increased production of free radicals and consequent oxidative stress. Moreover, a higher basal serum MPO activity in regular training group confirms the importance of activated neutrophils as a source of free radical generation in regular exercise [6, 27, 29]. Higher basal serum vitamin C level in regular training group can be probably explained by the fact that vitamin C is continuously mobilized from adrenal gland in response to chronic exercise-induced oxidative stress [36]. However, it is also reasonable to consider that different diet habits of professional athletes (increased consumption of fruit and vegetables) could contribute to higher levels of vitamin C.

## 5. Conclusion

According to results presented in the current study and available literature data it is clear that both acute and regular exercise increase the free radical production, leading to consequent oxidative stress. Vitamin C supplementation can significantly decrease serum MDA levels and suppress lipid peroxidation, thereby confirming vitamin C antioxidant capacity in exercise-induced oxidative stress.

On the other hand, vitamin C supplementation could not affect serum MPO activity, pointing that there is no effect on neutrophil inflammatory response both in acute and regular exercise. However, these results do not put into question the importance of vitamin C as an antioxidant, since activated neutrophils are only one of several sources of free radicals in different types of physical activity.

## Figures and Tables

**Table 1:** Serum oxidative stress markers and vitamin C status in experimental groups before vitamin C oral supplementation.

	Acute exercise (*n* = 30)	Regular training (*n* = 30)	*P* value
MDA (*µ*mol/L)	3.04 ± 1.48	4.26 ± 0.97	0.001
Vitamin C (*µ*mol/L)	55.4 ± 11.93	62.27 ± 9.68	0.017
Asc (*µ*mol/L)	24.13 ± 8.80	21.23 ± 7.57	0.181
DHA (*µ*mol/L)	31.60 ± 10.09	41.03 ± 8.76	<0.001
Ratio of DHA/Asc^‡^	1.62 ± 0.90	2.16 ± 1.02	0.022
MPO (U/L)^‡^	45.80 ± 26.89	68.83 ± 13.65	<0.001

Serum malondialdehyde (MDA), total vitamin C, ascorbic acid (Asc), dehydroascorbic acid (DHA), and myeloperoxidase activity (MPO) in acute exercise group were compared to values in regular training group before vitamin C oral supplementation. Differences between groups were tested by independent-samples *t*-test and Mann-Whitney test (‡), respectively.

**Table 2:** Influence of vitamin C supplementation on oxidative stress markers and vitamin C status in regular training group.

	Before supplementation	After supplementation	*P* value
MDA (*µ*mol/L)	4.26 ± 0.97	3.30 ± 0.65	<0.001
Vitamin C (*µ*mol/L)	62.27 ± 9.68	88.83 ± 16.85	<0.001
Asc (*µ*mol/L)	21.23 ± 7.57	31.1 ± 9.76	<0.001
DHA (*µ*mol/L)	41.03 ± 8.76	57.73 ± 10.11	<0.001
Ratio of DHA/Asc	2.16 ± 1.02	1.99 ± 0.53	0.14
MPO (U/L)	68.83 ± 13.65	71.07 ± 17.13	0.45

Serum malondialdehyde (MDA), total vitamin C, ascorbic acid (Asc), dehydroascorbic acid (DHA), and myeloperoxidase activity (MPO) were measured in 30 male athletes before and after 14 days of vitamin C oral supplementation (2 g/daily). Differences were tested by paired-samples *t*-test.
